# Supplementary material for: Snf2 Proteins Are Required to Generate Gamete Pronuclei in Tetrahymena thermophila
Source: Microorganisms. 2022 Dec 7;10(12):2426. doi: 10.3390/microorganisms10122426 (PMC9786623; doi:10.3390/microorganisms10122426)
Supplement: Supplementary file 1 [file microorganisms-10-02426-s001.zip › microorganisms-2042969-supplementary.pdf]

<u>SUPPLEMENTAL MATERIALS</u>
Supplemental figures

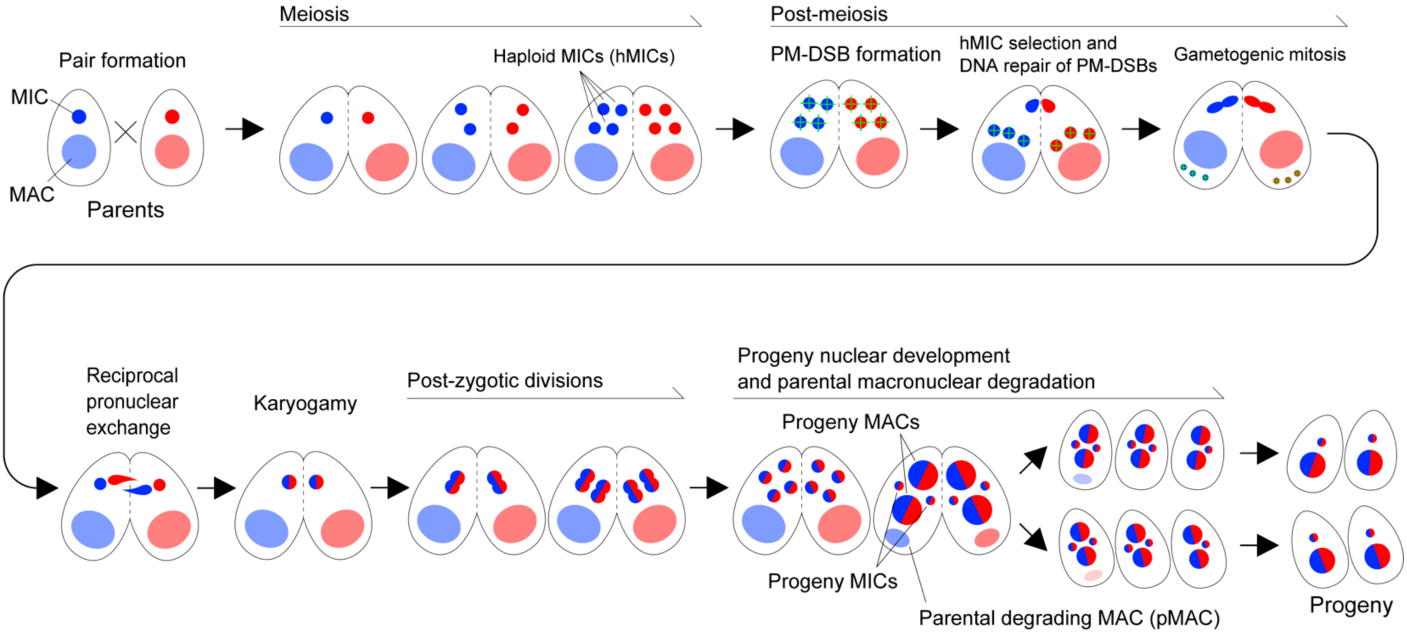

Supplemental figure S1 Representative nuclear events during *Tetrahymena* conjugation. **Pair formation:** When two starved cells of different mating types are mixed, they form a pair. **Meiosis:** After pair formation the MIC undergoes meiosis, resulting in four identical haploid MICs (hMICs). **Post-meiosis stage:** Post-meiotic DNA double-strand break (PM-DSB) formation takes place in all hMICs, cleaving their genome. As a result, γ-H2AX, an epigenetic marker for DNA lesions, appears. DNA lesions are repaired and γ-H2AX foci disappear in one of four hMICs. This is the selected hMIC, and the nucleus undergoes gametogenic mitosis, resulting in the production of two gametic pronuclei. **Reciprocal nuclear exchange and karyogamy:** One of the two gametic pronuclei is mutually exchanged with a partner cell. Then they fuse. **Post-zygotic mitosis:** The zygotic nucleus undergoes two rounds of mitosis, resulting in four zygotic nuclei. **Progeny nuclear development and parental MAC degradation:** Two of four zygotic nuclei locating in the anterior region of the cytoplasm become progeny MAC Anlagen. They swell and undergo differentiation involving genome rearrangement. The remaining two posterior nuclei become progeny MICs. Concurrently with progeny nuclear development, the parental MAC loses transcriptional activity and is eliminated from the cytoplasm via autophagy.

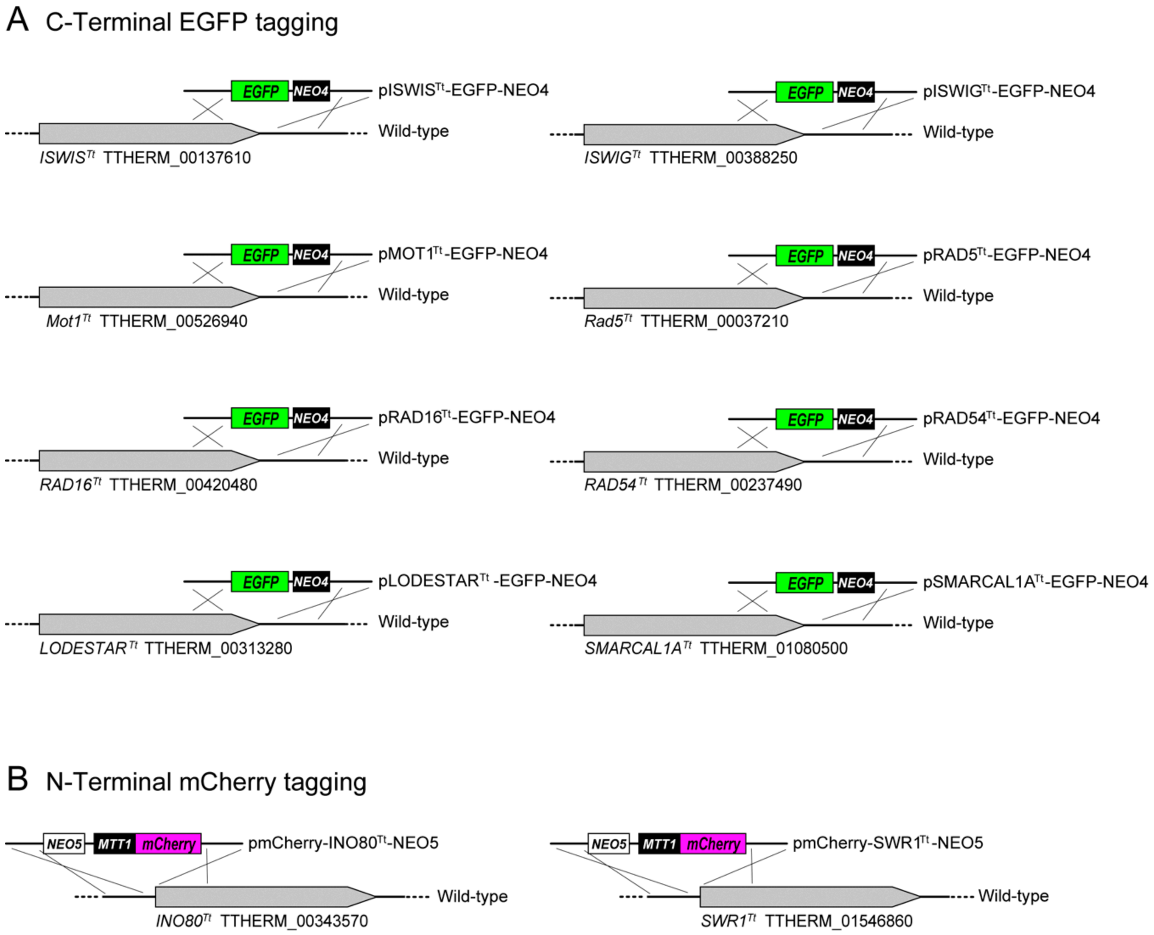

Supplemental figure S2 Cartoon showing the fluorescent-tag knock-in strategy.

"DNA preparation"

1 DNA fragment for transformation is amplified by PCR as in below.
  Volume: 320–400 µl (40–50 µl x 8 tubes)
  Enzyme: TaKaRa PrimeSTAR MAX DNA polymerase
  Primer: M13 forward primer and M13 reverse primer
  Thermal cycler: ABI9700 by the Life Technologies
  Thermal condition:
    Initial denature: 98°C, 50 sec
    Denature*: 98°C, 10 sec
    Annealing*: 50°C, 15 sec
    Elongation*: 72°C, 60 sec
    *From the denature to the elongation, are cycled 35 times.
    Additional elongation: 72°C, 120 sec

2 Amplified DNA is purified by the Monarch PCR & DNA Cleanup Kit (NEB).
    Tips for DNA purification.
    1) Amplified products with four tubes are gathered into one collection tube.
    2) Separately purchased Monarch Plasmid Miniprep Columns are applied for the DNA binding step.
    3) 50–80 µl of the DNA elution buffer per column is applied.
    As a result, 100–160 µl of DNA solution containing 25–50 µg of DNA for the transfection is obtained.

"Electroporation"

1 *Tetrahymena* cells are grown in 10 ml of SPP at 30°C with shaking overnight.
2 Cells are harvested by centrifugation at 300 *g* for 3 min, then the supernatant is discarded.
3 The collected cells are suspended with 10 ml of 10 mM Tris-HCl at pH 7.6.
4 The wash step (2–3) is repeated.
5 Washed cells are suspended in 10 ml of 10 mM Tris-HCl at pH 7.6 and are left at 30°C without shaking overnight
6 Cells are harvested by centrifugation at 300 *g* for 3 min, then the supernatant is discarded.
7 The collected cells are suspended with 10 ml of 10 mM Hepes-NaOH at pH 7.5. and left at least 5 min.
8 Cells are harvested by centrifugation at 300 *g* for 3 min, then the supernatant is discarded*.
  *At step 8, a small amount of buffer is left to become the total volume as 500 µl.
9 Immediately, the cell pellet is resuspended with the remaining buffer, then the cell suspension is transferred into a 1.5 ml tube.
10 Cells are collected at 1000 *g* for 2 min, then the supernatant is removed completely.
11 The purified DNA for the transfection is added, and the cell pellet is suspended completely by vertex for as short a time as possible.
12 The cell suspension with DNA for the transfection is transferred into an electroporation cuvette with a 2 mm gap.
13 Electroporation is carried out at 25 µF, 230 V, and 400Ω.
  *The pulsed-time and applied voltages are usually in the following ranges.
   Pulsed-time: 3.0–5.0 msec, Applied voltage: 220–235 V
14 After plus, the cell suspension is transferred into 40 ml of SPP and left at 30 °C for 3–4 h with shaking.
15* $CdCl_2$ is added (final concentration: 1 µg/ml) to activate the NEO4 cassette, then the cells are incubated at 30°C for 1 h.
  *This is an unnecessary step if NEO5 is used.
16 Add paromomycin sulfate to reach the concentration of 100 µg/ml, then the cells are transferred to two 96-well plates.
17 The plates are incubated at 30°C. Resistant cells will appear within 3 days.

Supplemental figure S3 The detailed procedure for DNA transfection into the MAC with electroporation. The procedure for DNA transfection into the MAC in starved cells is performed in a similar manner to the DNA transfection into conjugating cells.

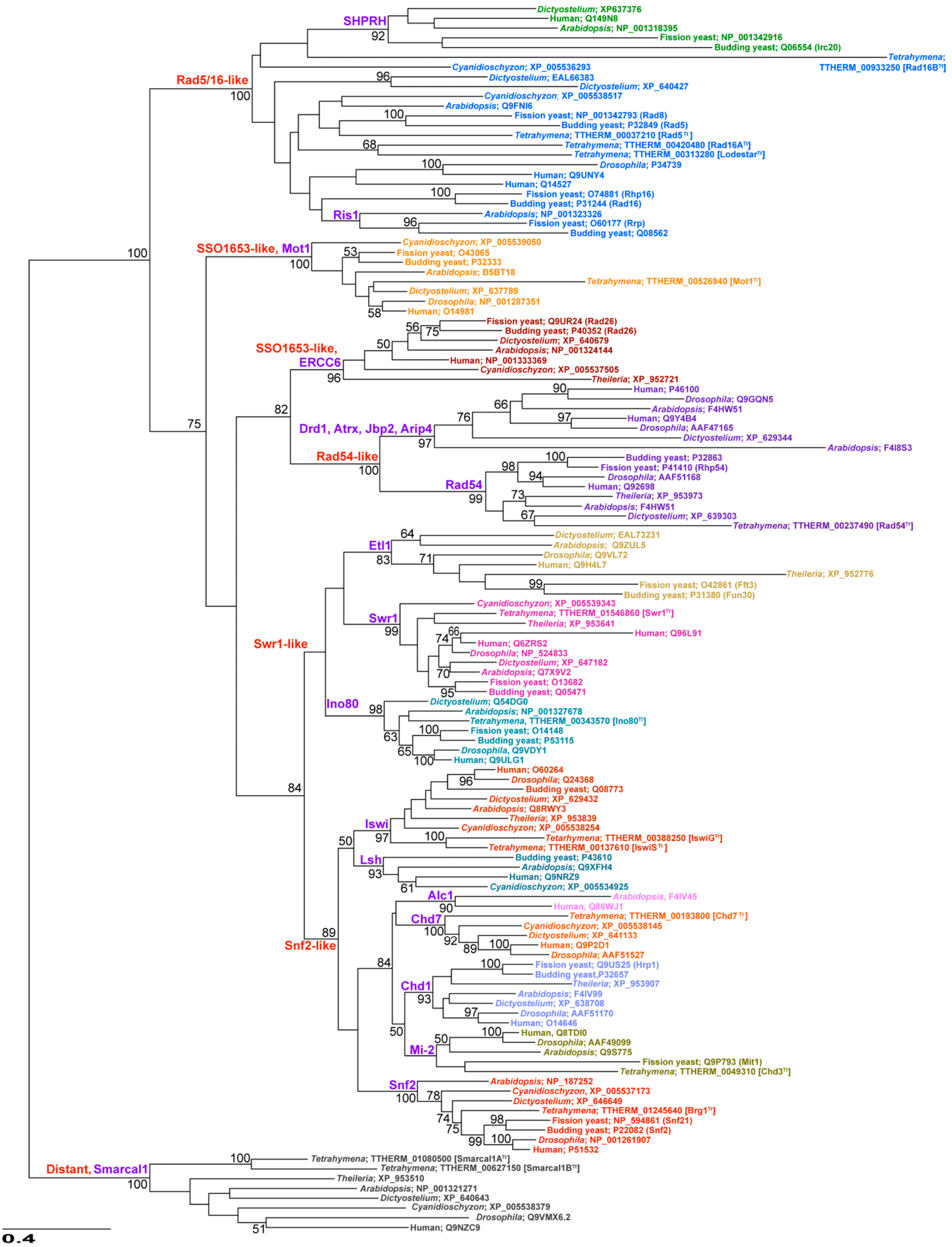

Supplemental figure S4 An unrooted ML phylogenetic tree based on conserved amino acid sequences corresponding to the SNF_N and HELICc domains. Groups and subfamilies are indicated in red and purple, respectively. Species and accession IDs are given at the branch ends, and protein names are given in parentheses. The name of Snf2 family proteins in *Tetrahymena* are given in brackets. Bootstrap values greater than 50 are shown at the branches.

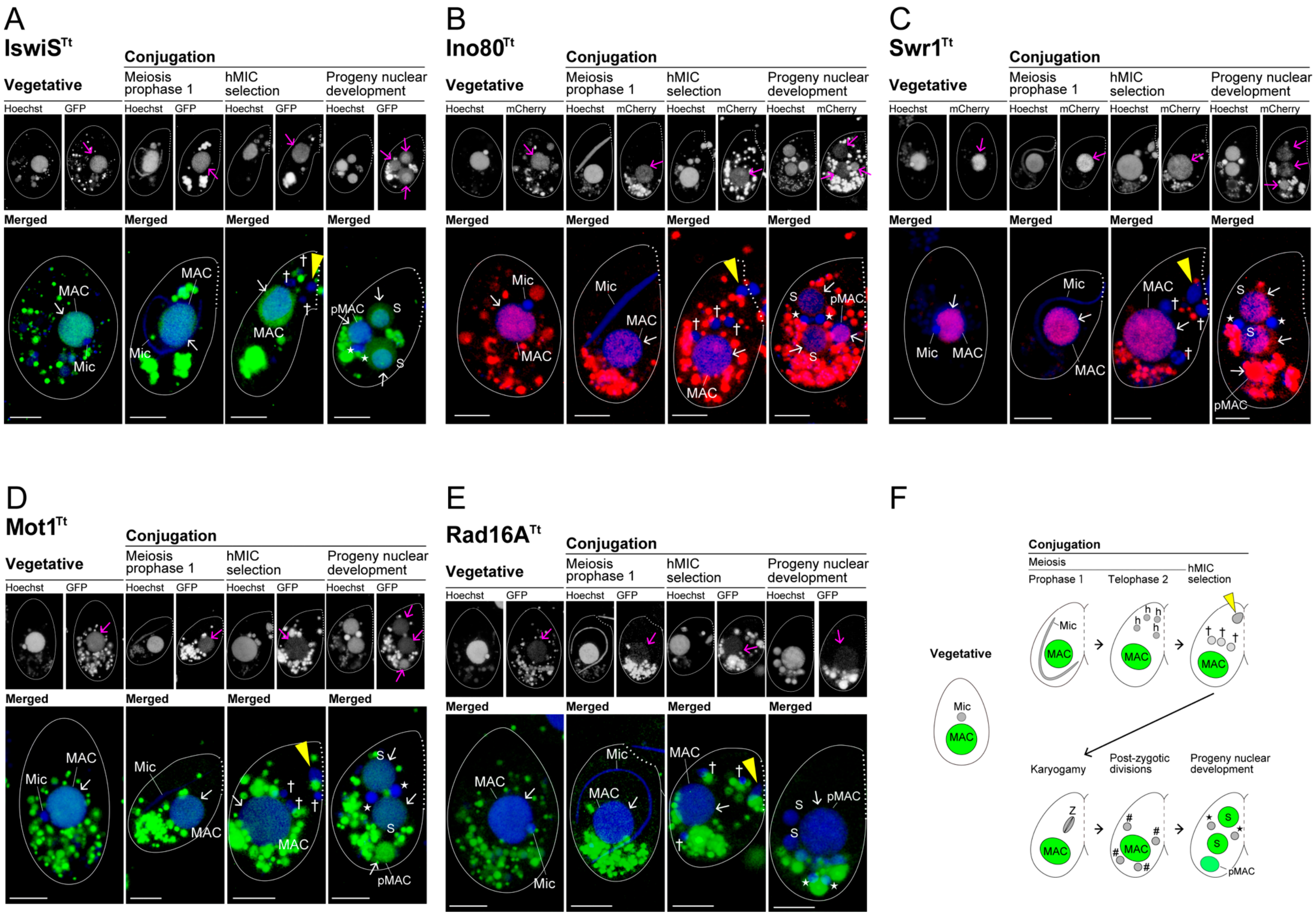

Supplemental figure S5 Subcellular localization of *Tetrahymena* Snf2 family proteins tagged with fluorescent proteins. Tagged proteins shown here exclusively localized to the MAC. **A)** C-terminally GFP-tagged IswiS[Tt]. **B)** N-terminally mCherry-tagged Ino80[Tt]. **C)** N-terminally mCherry-tagged Swr1[Tt]. **D)** C-terminally GFP-tagged Mot1[Tt]. **E)** C-terminally GFP-tagged Rad16A[Tt]. Arrows in the images for fluorescence localization indicate signals in nuclei. The scale bar denotes 10 µm. **F)** Schematic diagram of the subcellular localization of fluorescent signals. Triangle-the selected hMIC; †-unselected hMIC; Z-zygotic nuclei; #- zygotic nuclei undergoing post-zygotic nuclear divisions; S-progeny MAC Anlagen; star-progeny MIC; pMAC-degrading parental MAC.

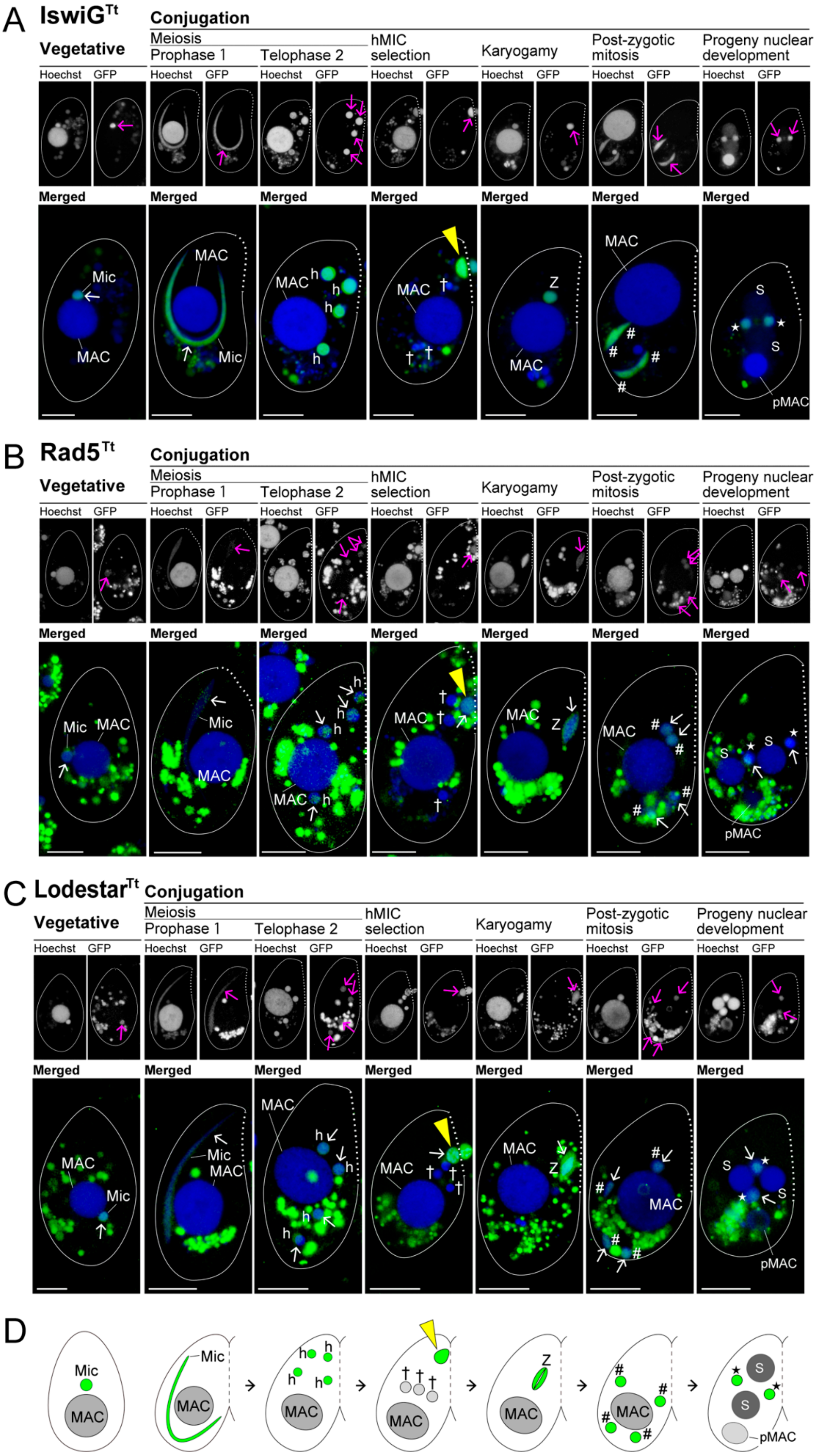

Supplemental figure S6 Subcellular localization of *Tetrahymena* Snf2 family proteins tagged with C-terminal GFP. Tagged proteins shown here exclusively localized to the MIC. **A)** C-terminally GFP-tagged IswiG^{Tt}. **B)** C-terminally GFP-tagged Rad5^{Tt}. **C)** C-terminally GFP-tagged Lodestar^{Tt}. **D)** The cartoon shows fluorescent signals. Triangle-the selected hMIC; †-unselected hMIC; Z-zygotic nuclei; #- zygotic nuclei undergoing post-zygotic nuclear divisions; S-progeny MAC Anlagen; star-progeny MIC; pMAC-degrading parental MAC.

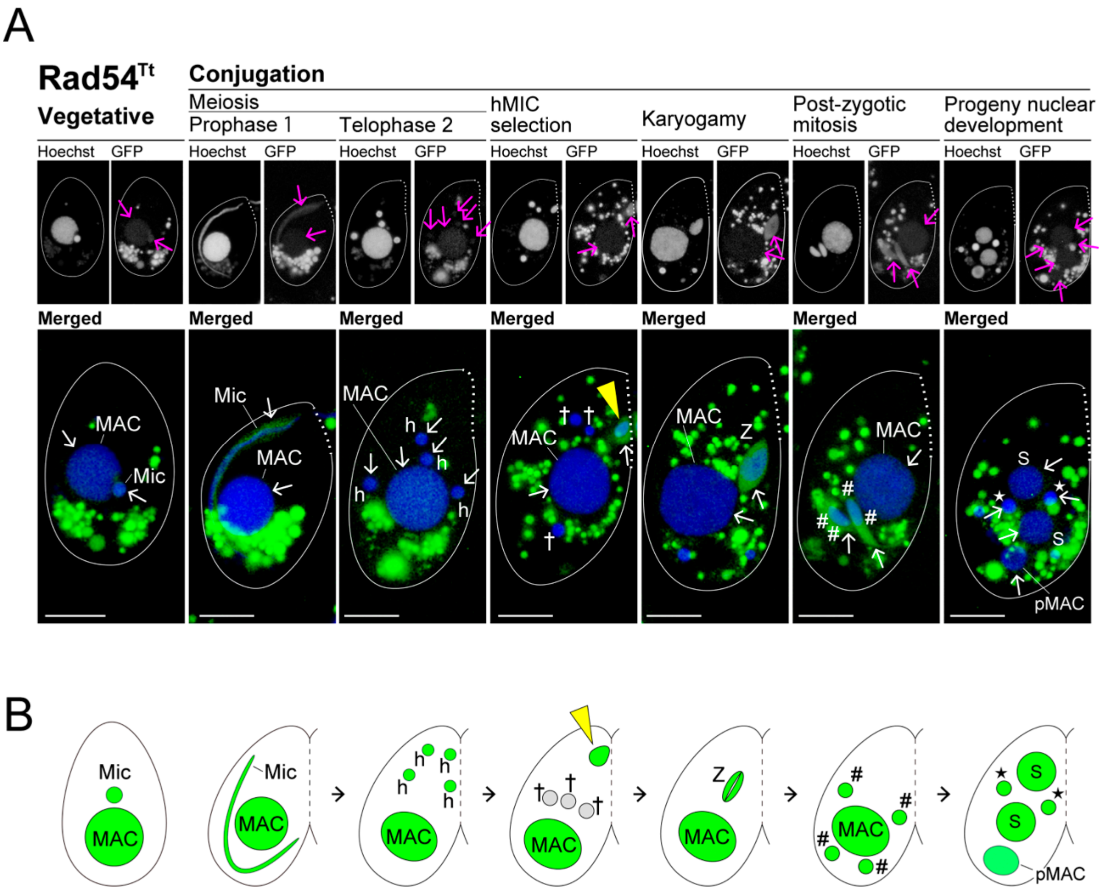

Supplemental figure S7 Subcellular localization of C-terminally GFP-tagged Rad54^{Tt} **A)** Rad54^{Tt}-EGFP appeared in both the MAC and MIC. **B)** Schematic diagram of the subcellular localization of fluorescent signals. Triangle-the selected hMIC; †-unselected hMIC; Z-zygotic nuclei; #- zygotic nuclei undergoing post-zygotic nuclear divisions; S-progeny MAC Anlagen; star-progeny MIC; pMAC-degrading parental MAC.

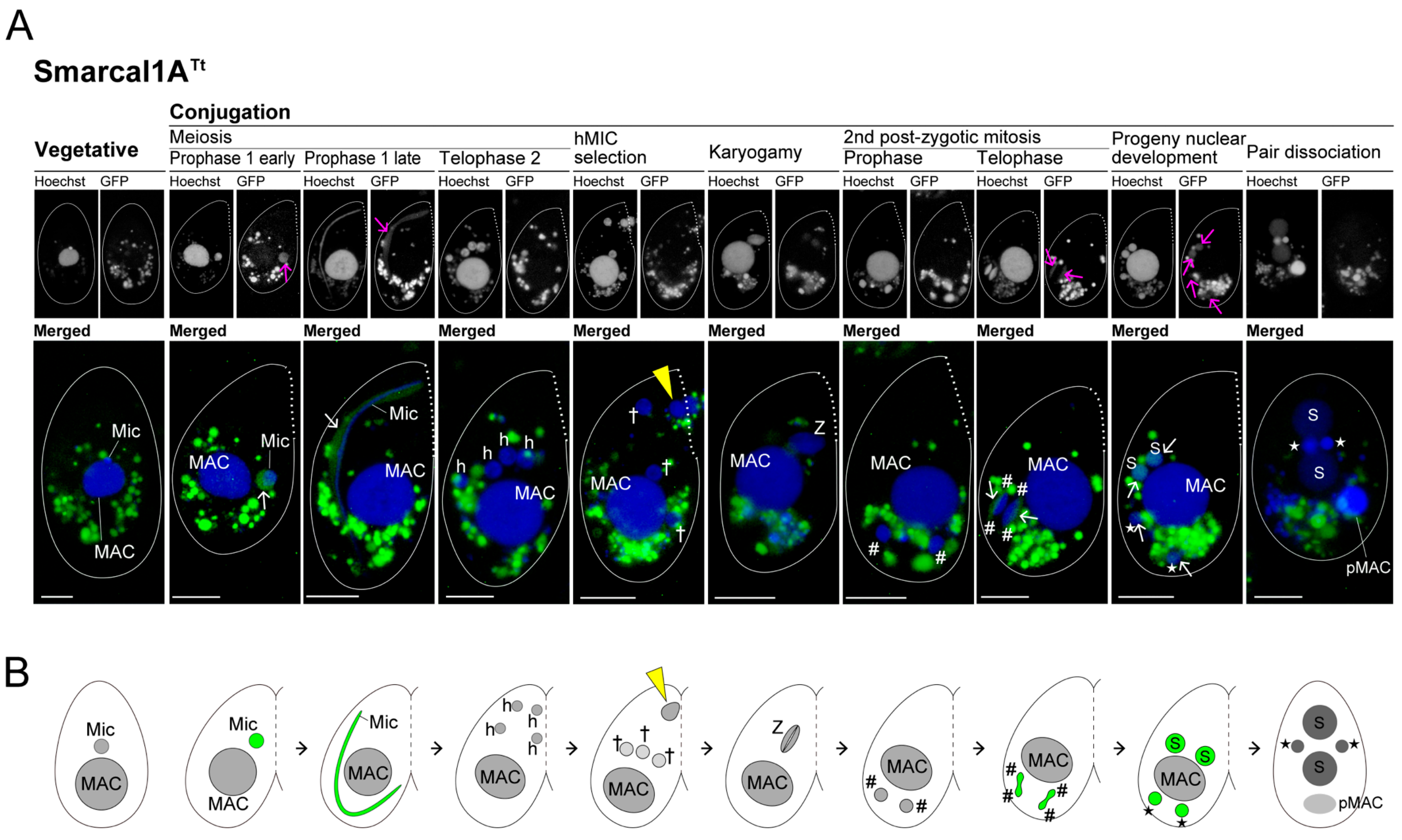

Supplemental figure S8 Subcellular localization of C-terminally GFP-tagged Smarcal1A^{Tt}. **A)** C-terminally GFP-tagged Smarcal1A^{Tt} appeared in the MIC undergoing meiosis anaphase 1 and zygotic nuclei undergoing 2nd post-zygotic mitosis. **B)** Schematic diagram of the subcellular localization of fluorescent signals. Triangle-the selected hMIC; †-unselected hMIC; Z-zygotic nuclei; #- zygotic nuclei undergoing post-zygotic nuclear divisions; S-progeny MAC Anlagen; star-progeny MIC; pMAC-degrading parental MAC.

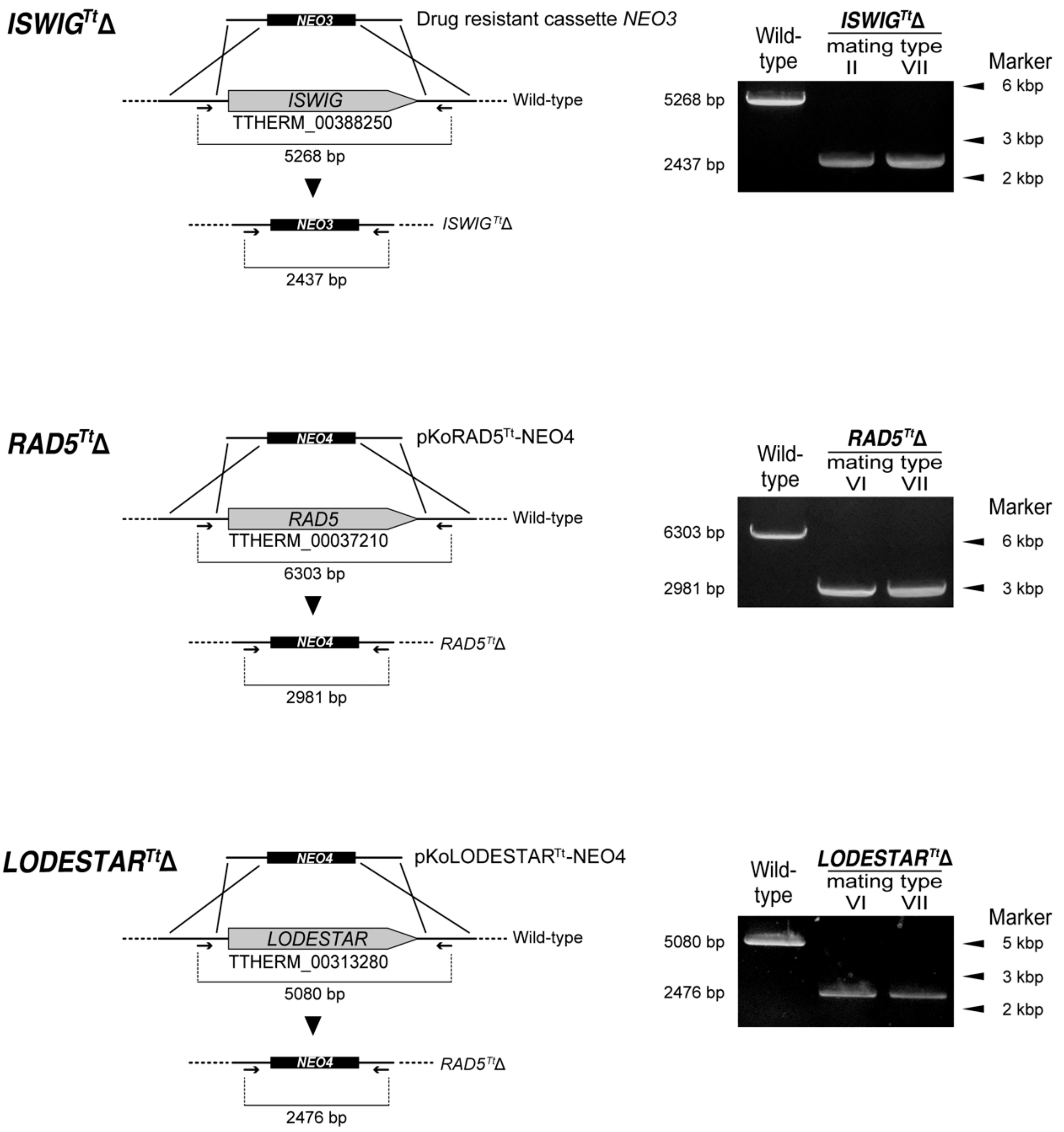

Supplemental figure S9 Generation of *ISWIG*^{Tt}Δ, *Lodestar*^{Tt}Δ, and *RAD5*^{Tt}Δ cells and PCR confirmation. Schematic diagrams for each knockout target gene show the following items. Upper-the knockout vector carrying the *NEO* cassette; Middle- the wild-type locus of the target gene. Lower-after homologous recombination. The primer sets, represented by arrows, are located upstream and downstream of the target gene locus. In the gel image, amplified PCR products from the wild-type genome are longer than those from knockout mutants because the *NEO* cassettes are shorter than genes encoding MIC localizing Snf2 family proteins.

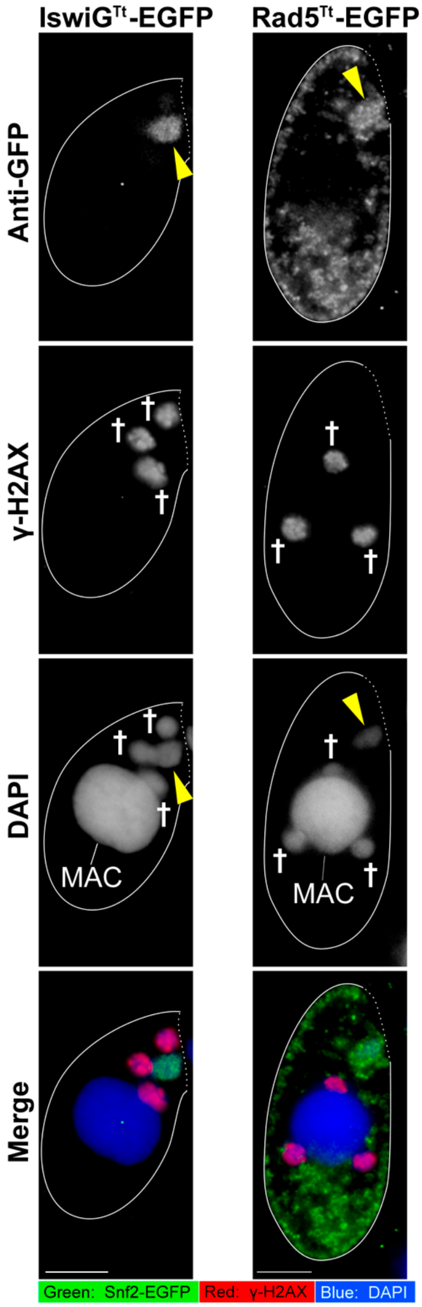

Supplemental figure S10 The IswiG^{Tt} and Rad5^{Tt} appeared in the selected hMIC in which γ-H2AX foci vanished. Triangle: selected hMIC, †: unselected hMIC. The scale bar denotes 10 μm.

Supplemental tables

Supplemental table S1. List of Snf2 genes found in *Tetrahymena* genome

TGD ID	Group	Subfamil	Name	Subcelluar localization	Tagging	Note
TTHERM_0124564	SNF2-like	Snf2	Brg1^{Tt}	MAC [46]	-	
TTHERM_0013761		Iswi	IswiS^{Tt}	MAC	C-terminal EGFP	
TTHERM_0038825		Iswi	IswiG^{Tt}	MIC, hMIC, selected hMIC, zygotic nuclei, progeny MIC	C-terminal EGFP	
TTHERM_0004931		Mi-2	Chd3^{Tt}	MAC [9]	-	
TTHERM_0019380		Chd7	Chd7^{Tt}	MAC [9]	-	
TTHERM_0154686	SWR1-like	Swr1	Swr1^{Tt}	MAC	N-terminal	
TTHERM_0034357		Ino80	Ino80^{Tt}	MAC	N-terminal	
TTHERM_0052694	SSO1653-like	Mot1	Mot1^{Tt}	MAC	C-terminal EGFP	
TTHERM_0023749	RAD54-like	Rad54	Rad54^{Tt}	MAC, MIC, hMIC, selected hMIC, zygotic nuclei, progeny MAC, and progeny MIC	C-terminal EGFP	
TTHERM_0003721	RAD5/16-like	Rad5/16	Rad5^{Tt}	MIC, hMIC, selected hMIC, zygotic nuclei, progeny MIC	C-terminal EGFP	
TTHERM_0042048		Rad5/16	Rad16A^{Tt}	MAC	C-terminal EGFP	
TTHERM_0093325		Rad5/16	Rad16B^{Tt}	Not examined	-	Expression is not active throughout lefe cycle.
TTHERM_0031328		Lodestar	Lodestar^{Tt}	MIC, hMIC, selected hMIC, zygotic nuclei, progeny MIC	C-terminal EGFP	
TTHERM_0108050	Distant	Smarcal1	Smarcal1A^{T}	MIC (meiosis 1) and zygotic nuclei after the anaphase of 2nd post zygotic mitosis	C-terminal EGFP	
TTHERM_0062715		Smarcal1	Smarcal1B^{Tt}	Not examined	-	Expression begins from late phase (after 8 hours post mixing) of conjugation.

Supplemental table S2. Primer sets for C-terminal EGFP-tagging and N-terminal mCherry-tagging

TGD ID	Name	Primer name*	Sequences#
TTHERM_00137610	IswiS^{Tt}	MACISWI-EGFP1F	TCCACCGCGGTGGCGGCCGCaattcagccatttacaataaatc
		MACISWI-EGFP2R	CCCTTAGAAACCATGGATCCatcacgctttaactttttttaagg
		MACISWI-EGFP5F	TCGATACCGTCGACCTCGAGacctgtaataaatagataaataattc
		MACISWI-EGFP6R	TATAGGGCGAATTGGGTACCtagaaggttttaggtcacaga
TTHERM_00388250	IswiG^{Tt}	MICISW2-EGFP1F	TCCACCGCGGTGGCGGCCGCaggaaaagcttggaattattg
		MICISW2-EGFP2R	CCCTTAGAAACCATGGATCCctttttaactattttatcaattttactaa
		MICISW2-EGFP5F	TCGATACCGTCGACCTCGAGtaaaactatttattagtgtccctc
		MICISW2-EGFP6R	TATAGGGCGAATTGGGTACCaataggcaatttgttatcttagtg
TTHERM_01546860	Swr1^{Tt}	SWR1-NmCherry1F	GGGAACAAAAGCTGGAGCTCtaaacctagttgatataaaccttcatctcc
		SWR1-NmCherry2R	TTTGAAGATATCAAGTCGACtttctttcataattaagaaatccttgactc
		SWR1-NmCherry5F	ATGAATTATATAAGGGATCCagttcggagtaggataaaaaccaaac
		SWR1-NmCherry6R	TATAGGGCGAATTGGGTACCtaaaccatttgattgcaggattcagc
TTHERM_00343570	Ino80^{Tt}	INO80-NmCherry1F	TCCACCGCGGTGGCGGCCGCtatgactttgtatactatgtatctcc
		INO80-NmCherry2R	TTTGAAGATATCAAGTCGACtgaataatctctctttatgaaagc
		INO80-NmCherry5F	ATGAATTATATAAGGGATCCatgctttggatgaagggttgt
		INO80-NmCherry6R	TATAGGGCGAATTGGGTACCacttattaagtgcgtgtttagagc
TTHERM_00526940	Mot1^{Tt}	MOT1-EGFP01F	TCCACCGCGGTGGCGGCCGCtcatcaatagtaagaactagatagatgg
		MOT1-EGFP02R	CCCTTAGAAACCATGGATCCatattctttatttatatcatctgtaaccc
		MOT1-EGFP05F	TCGATACCGTCGACCTCGAGtttgctttataataacttacctgcc
		MOT1-EGFP06R	TATAGGGCGAATTGGGTACCaagagcataagattctatttcttattagc
TTHERM_00237490	Rad54^{Tt}	RAD54_EGFP1F	TCCACCGCGGTGGCGGCCGCaatcagaatttgcaaagaattcg
		RAD54_EGFP2R	CCCTTAGAAACCATGGATCCtttttttaatttttaagtatatttttggc
		RAD54_EGFP5F	TCGATACCGTCGACCTCGAGactaaatcaaaaaagatatttaattattcg
		RAD54_EGFP6R	TATAGGGCGAATTGGGTACCttgattttgacttgaaatattactgg
TTHERM_00037210	Rad5^{Tt}	Rad5-EGFP1F	TCCACCGCGGTGGCGGCCGCaggaaatacaggttaacaaaacc
		Rad5-EGFP2R	CCCTTAGAAACCATGGATCCatatgttgacattagatatttaaagtgc
		Rad5-EGFP5F	TCGATACCGTCGACCTCGAGactaatatcaatctattccttaaaacat
		Rad5-EGFP6R	ATAGGGCGAATTGGGTACCtcaatcataaataatctttagtgctc
TTHERM_00420480	Rad16A^{Tt}	RAD16A-EGFP1F	TCCACCGCGGTGGCGGCCGCttattagtaagcttaataaccttcagatg
		RAD16A-EGFP2R	CCCTTAGAAACCATGGATCCattcaatatctgttataagaattcttttgc
		RAD16A-EGFP5F	TCGATACCGTCGACCTCGAGtaatttcaataataattttatatttaacctaaaatg
		RAD16A-EGFP6R	TATAGGGCGAATTGGGTACCtactgaaaatcaagagatattttgtgc
TTHERM_00313280	Lodestar^{Tt}	Lodestar-EGFP1F	TCCACCGCGGTGGCGGCCGCttagatggtgtctcactggtactcc
		Lodestar-EGFP2R	CCCTTAGAAACCATGGATCCtaatttatcttcagctctaaattatttttatgg
		Lodestar-EGFP5F	TCGATACCGTCGACCTCGAGaacaaattaaaaaataaaattttatgcg
		Lodestar-EGFP6R	TATAGGGCGAATTGGGTACCttaattcaagttatcctaaatgttgtc
TTHERM_01080500	Smarcal1A^{Tt}	Smarcal1A-EGFP1F	TCCACCGCGGTGGCGGCCGCaaatggctcttcatcaatgttttctgc
		Smarcal1A-EGFP2R	CCCTTAGAAACCATGGATCCaaaatgctactttgctaatttcaaaagtg
		Smarcal1A-EGFP5F	TCGATACCGTCGACCTCGAGtttggatttaaaacatatttaattggtc
		Smarcal1A-EGFP6R	TATAGGGCGAATTGGGTACCagatataaacatctgttttgagatgc

*: Amplified products from EGFP/NmCherry1F-2R and EGFP/NmCherry5F-6R are 5' and 3' regions giving homologous recombination, respectively.

$: Capital letters correspond to the region for homologous recombination with plasmids, and lowercase letters correspond to the region matching to the *Tetrahymena* genome.

Supplemental table S3. Antibodies used in the indirect immunofluorescence observations

Figure No.	Primary antibody				Secondary antibody				
	Epitope	Dilution	Cat. No.	Company	Epitope	Label	Dilution	Cat. No.	Company
4	γ-H2AX (H2AX S139ph)	1:1000	613401	BioLegend	Mouse IgG (H+L)	FITC	1:500	31541	Thermo Scientific
5	H3K9ac	1:1000	MABI0305S	MBL	Mouse IgG (H+L)	Alexa Fluor® 568	1:500	ab175700	Abcam
	H3K18ac	1:1000	ab40888	Abcam	Rabbit IgG (H+L)	Rhodamine	1:500	31686	Thermo Scientific
	H3K56ac	1:1000	39282	Activ	Rabbit IgG (H+L)	Rhodamine	1:500	31686	Thermo Scientific
S9	GFP	1:1000	598	MBL	Rabbit IgG	DyLight™ 488	1:500	406404	BioLegend
	γ-H2AX (H2AX S139ph)	1:1000	613401	BioLegend	Mouse IgG (H+L)	Alexa Fluor® 568	1:500	ab175700	Abcam

Supplemental table S4. Primer sets for somatic gene knockout plasmids

Target	TGD ID	Primer name*	Sequences
LODESTAR^{Tt}	TTHERM_00313280	LodestarKO_pNeo4_1F	TCCACCGCGGTGGCGGCCGCttaattcaagttatcctaaatgttgtc
		LodestarKO_pNeo4_2R	TATTTTTTAGAAATAAATTGTaacaaattaaaaaataaaattttatgcgc
		LodestarKO_pNeo4_5F	ACTGGAAAAATGCAGCCCGGGagtgaagtcaaataaatcaatatcgtg
		LodestarKO_pNeo4_6R	TATAGGGCGAATTGGGTACCtcttttttgaataccataccaagag
RAD5^{Tt}	TTHERM_00037210	Rad5KO_pNeo4_1F	CTCCACCGCGGTGGCGGCCGCtcaatcataaataatctttagtgctc
		Rad5KO_pNeo4_2R	TATTTTTTAGAAATAAATTGTactaatatcaatctattccttaaaacat
		Rad5KO_pNeo4_5F	ACTGGAAAAATGCAGCCCGGGttgagtaataaaagatatgcaaaatctatg
		Rad5KO_pNeo4_6R	CTATAGGGCGAATTGGGTACCatattgttaaattcttccactaaacctg
RAD54^{Tt}	TTHERM_00237490	Rad54KO_pNeo4_1F	CTCCACCGCGGTGGCGGCCGCttgattttgacttgaaatattactgg
		Rad54KO_pNeo4_2R	TATTTTTTAGAAATAAATTGTactaaatcaaaaaagatatttaattattcg
		Rad54KO_pNeo4_5F	ACTGGAAAAATGCAGCCCGGGtgatttattatttactttaaatctatcc
		Rad54KO_pNeo4_6R	CTATAGGGCGAATTGGGTACCagtatagatgaggcttttgttg

*: Amplified products from pNoe4_1F-2R and pNoe4_5F-6R are 5' and 3' regions giving homologous recombination, respectively.

Supplemental table S5. Primer sets for PCR evaluating somatic gene replacement

Target	TGD ID	Primer name	Sequences
LODESTAR^{Tt}	TTHERM_00313280	LodestarKO_SG-F	tagaatgttattactcttcattggc
		LodestarKO_SG-R	caatttatctaaattttgatacatagctcg
RAD5^{Tt}	TTHERM_00037210	Rad5KO_SG-F	tagaatgttattactcttcattggc
		Rad5KO_SG-R	caatttatctaaattttgatacatagctcg
RAD54^{Tt}	TTHERM_00237490	Rad54KO_SG-F	gcagtaatcagtgatggttcttacg
		Rad54KO_SG-R	ttatggagaattgcttgatatctgc
ISWIG^{Tt}	TTHERM_00388250	IswiG_Neo3KO_SG-F	ttgaatatggaggtttagcttgc
		IswiG_Neo3KO_SG-R	taaaaggtctgcgcatagttatagc
